# Discrepancy between catheter and doppler gradients immediately post transcatheter aortic valve replacement in an underexpanded prosthesis

**DOI:** 10.1002/ccr3.1547

**Published:** 2018-04-22

**Authors:** Imad Hameedullah, Elnazeer O. Ahmed, Abdalla Alzoobiy, Osama Elkhateeb

**Affiliations:** ^1^ Cardiovascular Research Unit King Abdullah Medical City Makkah Saudi Arabia; ^2^ Department of Cardiac Surgery King Abdullah Medical City Makkah Saudi Arabia; ^3^ Department of Cardiology King Abdullah Medical City Makkah Saudi Arabia; ^4^ Department of Cardiology Dalhousie University Halifax Nova Scotia Canada

**Keywords:** Aortic valve disease percutaneous, transcatheter valve implantation

## Abstract

Underexpansion of an aortic bioprosthetic valve is common after transcatheter aortic valve replacement (TAVR). Significant discrepancy between invasive hemodynamic gradients and echocardiographic Doppler gradients in an underexpanded bioprosthesis can be attributed to pressure recovery phenomenon. This case emphasizes the importance of echocardiographic guidance in implantation and assessment of bioprosthetic valve during TAVR.

## Introduction

Transcatheter aortic valve replacement (TAVR) is the procedure of choice for patients with high and intermediate risk profile. The success of the procedure is dependent on correct position of the prosthesis, appropriate expansion of the valve, improvement in hemodynamics including mean gradient of less than 20 mmHg, and minimal aortic insufficiency.

Bicuspid aortic valves (BAV) are commonly observed in patients above 70 years old and are amenable to TAVR. TAVR in BAV patients is challenging, and this patient population was excluded in initial TAVR trials. BAV have significant calcification, and their abnormal shape contributes to significant aortic insufficiency (AI). Moreover, an elliptical shape of the bioprosthesis during or after implant may lead to residual gradient across the valve.

An elliptical or oval shape of the bioprosthesis may occur post‐TAVR in patients with BAV or severe aortic valve calcification. Oval shape morphologies have been reported in both manufacturers of the prosthetic valve 1. Oval shape lodgment of the bioprosthesis affecting hemodynamic function is a novel finding of this case report.

Valve assessment is performed via multiple modalities such as echocardiography, cardiac computed tomography (CT), and most accurately, by catheter hemodynamic assessment of the aortic valve in the catheterization laboratory. Invasive methods and noninvasive methods have shown to be well correlated 2. There are reports of discrepancy between invasive catheter‐based and echocardiographic Doppler gradients which can be attributed to different mechanisms including pressure recovery phenomenon.

## Case Report

Our patient is an 80‐year‐old female with no previous history of cardiac disease. She presented with 2 months of worsening dyspnea. Her past medical history was significant for hypertension and severe osteoarthritis; the latter contributed to her limited mobility. On examination, her pulse was regular with delayed upstroke. Jugular venous pressure was elevated, and there were bilateral crackles on chest auscultation. There was normal first heart sound, decreased second heart sound, and loud fourth heart sound. There was an ejection systolic murmur in keeping with significant aortic stenosis. Electrocardiogram showed sinus rhythm. TTE showed severe calcific aortic stenosis. Aortic stenosis was confirmed with a peak gradient of 138 mmHg and mean gradient of 76 mmHg. The ejection fraction of the left ventricle was normal at 55% with mild mitral regurgitation.

**Figure 1:**
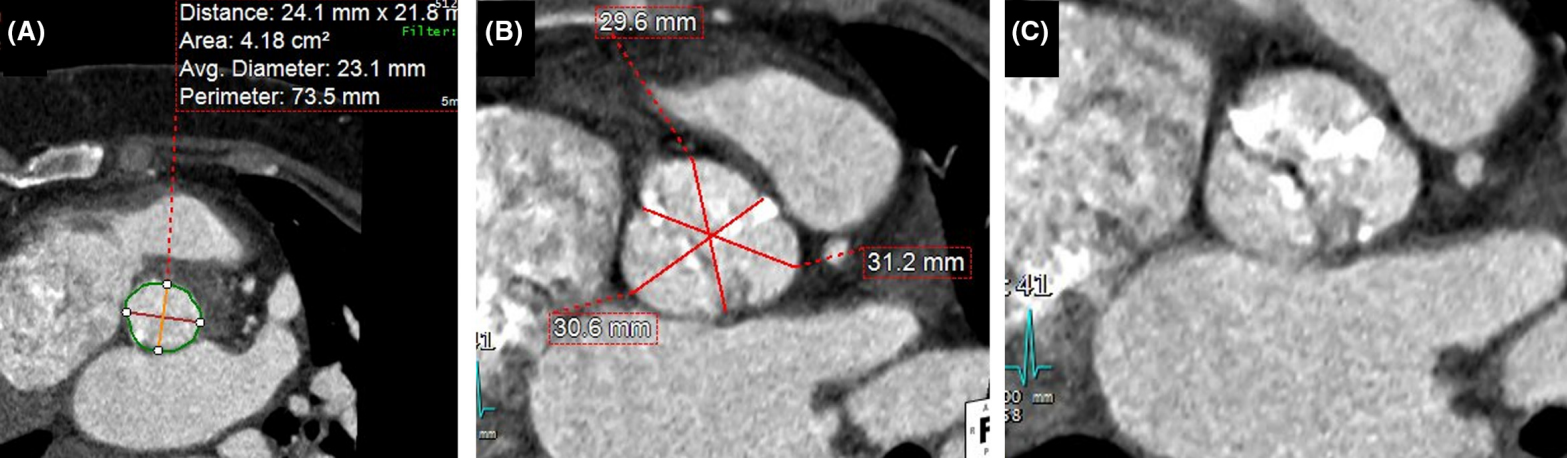
Cardiac CT images of valve preprocedure. (A) Measurements at annulus level. (B) Measurements at sinus level. (C) The bicuspid morphology of valve leaflets.

Cardiac CT confirmed presence of severe calcification and potentially functional BAV. The mean annular diameter was 23 mm, perimeter of 73.5 mm, annular area 4.18 cm^2^, sinus average diameter of 30 mm, and ascending aorta diameter was 29 mm (Fig. 1).

The calculated society of thoracic surgery (STS) score was 5.5 with Euroscore of 4.2. Multidisciplinary team meeting advocated for patient to undergo TAVR due to her frailty and poor mobility. Her advanced age and weak candidacy for surgery supported TAVR decision, and patient consent was obtained.

**Figure 2:**
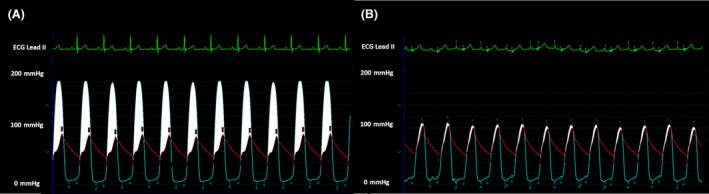
Catheter hemodynamic gradients pre (A) and post (B) TAVR showed improvements in pressure gradients to 5 mmHg.

**Figure 3:**
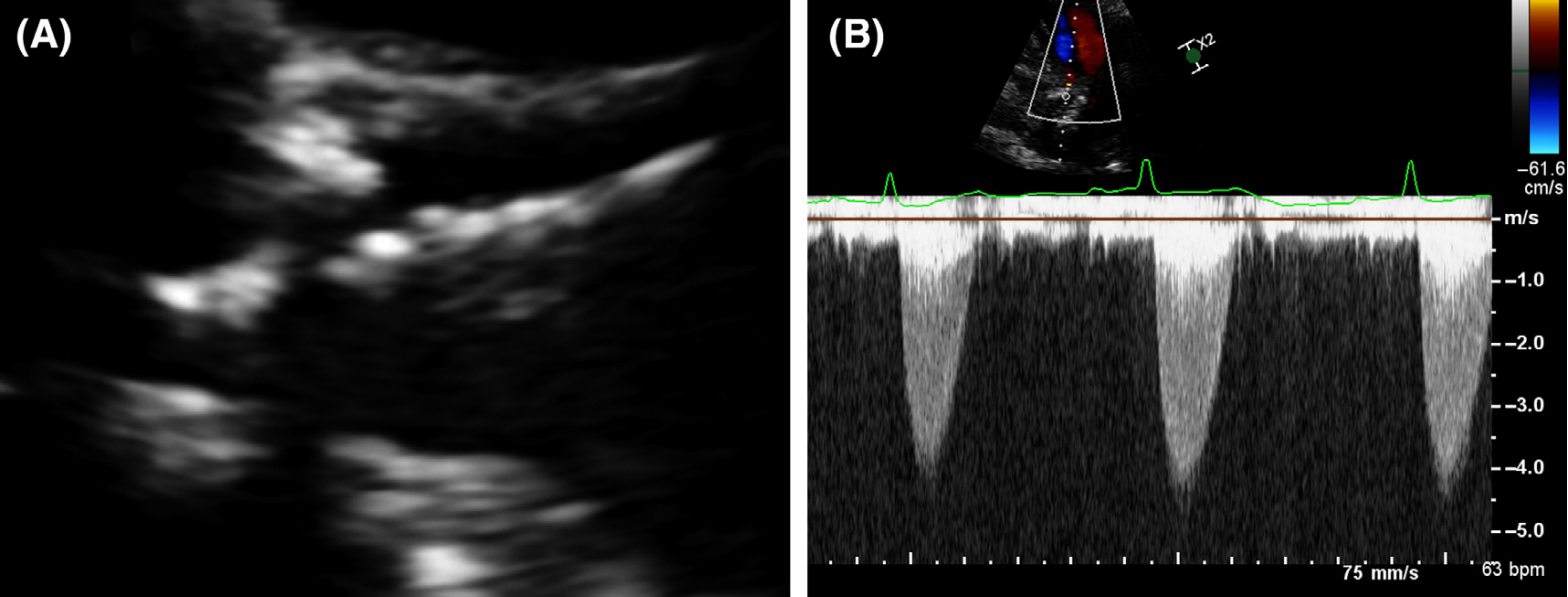
Post‐TAVR echocardiographic images and gradients. (A) 2D long‐access view transthoracic echocardiographic of bioprosthesis shows underexpansion of valve. (B) Doppler gradient was significant at mean/peak gradients of 42/72 mmHg.

**Figure 4:**
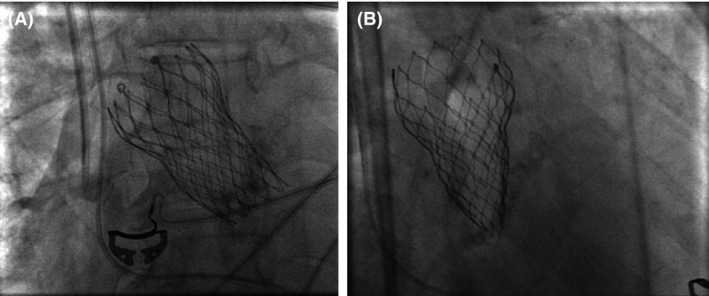
Fluoroscopic images of valve post‐TAVR. (A) LAO caudal 10 view shows well‐expanded valve. (B) Orthogonal RAO 48 view shows gross underexpansion of the bioprosthesis. LAO, left anterior oblique fluoroscopy view; RAO, right anterior oblique fluoroscopy view.

Under conscious sedation and through femoral approach, preclosure was achieved using two ProGlide (Abott Laboratories, Chicago, IL) closure devices. Temporary pacing wire was placed in the right ventricle. Predilatation with 20 mm by 4‐cm balloon enacted using rapid pacing technique. A 26 CoreValve Evolut R was advanced through the right femoral artery and implanted at the aortic valve tubular junction. Final invasive hemodynamic data confirmed minimal gradient across the valve of 5 mmHg (Fig. 2). There was no aortic insufficiency by fluoroscopy. Femoral sheath was removed, and the femoral artery was closed with the preimplanted ProGlide sutures. Patient was hemodynamically stable and transferred to the coronary care unit for observation. Routine postprocedure TTE showed significant gradients by Doppler of mean/peak 40/70 with gross underexpansion of the valve (Fig. 3). Subsequently, perpendicular fluoroscopic views confirmed underexpansion of the valve (Fig. 4). Patient had no heart failure, no arrhythmia, and asymptomatic. After discussion and consent, patient was taken back to the cath laboratory and the bioprosthetic valve was postdilated with multiple inflations of 22 mm by 4‐cm Z‐Med balloon (B. Braun Interventional Systems Inc., Bethlehem, PA) with improvements in Doppler gradients. The final mean/peak gradient by TTE Doppler was 18/35 mmHg.

Patient did well postprocedure with no further problems. TTE performed prior to discharge showed mean/peak gradient of 18/35 mmHg. Patient continued to do well with a dramatic improvement in her symptoms and functional class. A follow‐up TTE at six moths showed well functioning bioprosthetic valve with mean/peak gradient of 9/22 mmHg and no aortic regurgitation.

## Discussion

Underexpansion of an aortic bioprosthetic valve is common after TAVR especially in self‐expandable bioprosthetics. However, it appears that there are no significant hemodynamic or structural squealae because of this at least in the short term. Jilahawi et al. 2 reported incomplete expansion of CoreValve in 54% patients, but with no significant effects on peak or mean valve gradients nor any degree of aortic regurgitation.

In vitro studies showed that the effective circular dimension for the valve to function normal should be in a factor of <0.5. Moreover, an eccentricity of an elliptical valve of larger that 0.5 is associated with backflow leakage and increased stress on the leaflets 3.

In our patient, we described an underexpansion of CoreValve bioprosthesis in a severely calcified BAV despite predilatation. The underexpansion leads to significant Doppler gradients across the bioprosthetic valve despite near normal catheter gradients. The discrepancy between TTE and catheter gradients is not well understood. However, a potential explanation for this is pressure recovery phenomenon. Pressure recovery phenomenon occurs because of the downstream increase in the pressure because of the conversion of the kinetic energy to potential energy. These conversions result in overestimation of Doppler gradients as compared to catheter gradients. Valve morphology contributes to inflow and outflow gradients and the phenomenon of pressure recovery. It is more pronounced in tunnel‐like stenosis, which is similar to an underexpanded bioprosthesis as in our case. It can also occur in hypertrophic cardiomyopathy, surgically implanted prosthetic valves, and subvalvular or supravalvular stenoses 4.

A similar discrepancy published by Panoulas et al. 1 in a case with direct flow valve and felt to be secondary to pressure recovery and the nonflat velocity profile of the valve.

Experimental models in mechanical valves showed up to 40 mmHg difference between catheter‐based and Doppler gradients 5.

Furthermore, aorta size <30 mm can cause and exaggerate pressure recovery phenomenon 6. Our patient had an ascending aorta diameter of 29 mm, a possible cause of the gradients discrepancy.

Erroneous reading and equipment malfunction are another potential cause for the observed discrepancy. However, we are meticulous about zeroing transducers before each measurement especially in TAVR cases. In addition, the hemodynamic tracing (Fig. 2) showed valid wave forms with no evidence of pressure dampening.

Discrepancy between catheter and Doppler gradients continued to be there on the subsequent procedure. It was confirmed using simultaneous ventricular and femoral pressure due to single femoral puncture to minimize complications. This makes bioprosthetic valve recoil as a cause of this discrepancy less likely.

Underexpansion of bioprosthetic aortic valve can result in poor gradients in vitro. It is well documented at least in vitro that underexpansion of the bioprosthesis can affect function of the valve. Increased ovality of valve can also result in higher pressure gradients and regurgitant volume 4. Moreover, it may lead to localized high stress regions within the valve leaflets itself 7 and accumulating detrimental effects on the long‐term function of the bioprosthetic valve.

## Conclusion

This case emphasized the importance of echocardiographic guidance in implantation and assessment of bioprosthetic valve during TAVR. In addition, the need for echocardiographic Doppler measurements during procedure, and full echocardiographic study postprocedure for complete heart hemodynamic evaluation is warranted. Furthermore, orthogonal and diverse planes of fluoroscopic imaging in the catheterization laboratory aid in assuring full expansion of the bioprosthesis, especially in heavily calcified and bicuspid aortic valves. Gradient discrepancy between TTE and catheter measurements during TAVR may occur due to pressure recovery phenomenon and should be considered.

## Authorship

IH: contributed to analysis of hemodynamic data and heavily editing the manuscript. EO and AA: contributed to caring of the patient and involved in the procedure in addition to critically revised the manuscript. OE: contributed to performing the procedure and caring for the patient in addition to drafting and writing the manuscript and reviewing the literature.

## Conflict of Interest

None declared.
